# Removal of Per- and Polyfluoroalkyl Substances Using Commercially Available Sorbents

**DOI:** 10.3390/ma18061299

**Published:** 2025-03-15

**Authors:** Zhiming Zhang, Sevda Joudiazar, Anshuman Satpathy, Eustace Fernando, Roxana Rahmati, Junchul Kim, Giacomo de Falco, Rupali Datta, Dibyendu Sarkar

**Affiliations:** 1 Department of Civil and Environmental Engineering, Rowan University, Glassboro, NJ 08028, USA; zhangz@rowan.edu; 2 Department of Civil, Environmental and Ocean Engineering, Stevens Institute of Technology, Hoboken, NJ 07030, USA; sjoudiaz@stevens.edu (S.J.); satpathyanshuman92@gmail.com (A.S.); eustace6192@gmail.com (E.F.); rrahmati@stevens.edu (R.R.); 3 Tetra Tech, Inc., King of Prussia, PA 19406, USA; jc.kim@tetratech.com; 4 New York City Department of Environmental Protection, New York City, NY 11368, USA; 5 Department of Biological Sciences, Michigan Technological University, Houghton, MI 49931, USA; rupdatta@mtu.edu

**Keywords:** per- and polyfluoroalkyl substances (PFAS), adsorption, remediation, organoclays, modified organoclays, reactive media

## Abstract

Per- and polyfluoroalkyl substances (PFAS) are persistent organic pollutants of growing environmental and human health concern, widely detected across various environmental compartments. Effective remediation strategies are essential to mitigate their widespread impacts. This study compared the performance of two types of commercially available sorbent materials, granular activated carbon (GAC, Filtrasorb-400) and organoclays (OC-200, and modified organoclays Fluoro-sorb-100 and Fluoro-sorb-200) for the removal of three representative PFAS compounds: perfluorooctanoic acid (PFOA), perfluorononanoic acid (PFNA), and perfluorooctane sulfonic acid (PFOS) from water. Both organoclays and modified organoclays outperformed GAC, likely due to electrostatic interactions between the anionic PFAS compounds and the cationic functional groups of the modified organoclays. A pseudo-second-order kinetic model best described the rapid sorption kinetics of PFOA, PFNA, and PFOS. For PFOA, OC-200 demonstrated the highest adsorption capacities (q_max_ = 47.17 µg/g). For PFNA and PFOS, Fluoro-sorb-100 was the most effective sorbent, with q_max_ values at 99.01 µg/g and 65.79 µg/g, respectively. Desorption studies indicated that the sorption of the three PFAS compounds on these commercially available sorbents was largely irreversible. This study highlights the effectiveness and sorption capacities of different types of commercial sorbents for PFAS removal and offers valuable insights into the selection of reactive media for PFAS removal from water under environmentally relevant conditions.

## 1. Introduction

Per- and polyfluoroalkyl substances (PFAS) are a class of synthetic chemicals characterized by aliphatic carbon chains, where hydrogen atoms are partially (polyfluoroalkyl substances) or completely (perfluoroalkyl substances) substituted with fluorine atoms [1]. The stability conferred by the strong carbon–fluorine bonds makes PFAS highly persistent in the environment. Over the past few decades, their extensive use across various industrial applications has resulted in widespread environmental contamination, with detectable levels in wildlife and humans. Exposure to PFAS has been associated with numerous adverse health outcomes, including thyroid disorders, liver and kidney damage, reproductive dysfunction, and certain types of cancer [2]. Given their toxicity and environmental persistence, the development and implementation of effective remediation strategies for PFAS removal from environmental matrices are urgently required.

Several remediation strategies, including biological remediation [3], electrochemical oxidation [4], nanofiltration [5,6], and adsorption [7,8], have been recently explored for the removal of PFAS from the environment. However, the economic and environmental feasibility of many PFAS remediation techniques is not optimal. High energy demands, substantial capital investments, and the potential for considerable waste generation are key factors limiting their broader application [9]. Among all these techniques, adsorption stands out as a particularly promising method for PFAS removal from water, owing to its cost-effectiveness and operational simplicity [7,10,11]. Adsorption can be utilized in the in situ construction of permeable reactive barriers (PRBs) at PFAS-contaminated groundwater sites [12,13]. A critical factor in PRB design and operation is the selection of suitable reactive media [14]. The choice of reactive media not only affects the efficiency of contaminant removal but also impacts the longevity and maintenance demands of the PRB system [15]. Commercially available sorbents and reactive media are preferred due to their widespread availability, established performance, and cost-effectiveness, particularly for large-scale in situ remediation efforts, such as PRBs for contaminated groundwater treatment.

Granular activated carbon (GAC) is widely used as a commercially available material for the removal of organic and inorganic contaminants, acting as an effective sorbent [16,17]. A large specific surface area and porous structure make GAC highly suitable for applications in groundwater, industrial wastewater, and drinking water treatments [18,19,20,21]. Studies have demonstrated the effectiveness of GAC in removing PFAS from contaminated water. However, the adsorption of anionic PFAS species onto GAC is largely driven by non-specific interactions and is strongly influenced by the hydrophobicity of the PFAS species, such as chain length [22]. This renders GAC less effective for the removal of short-chain PFAS.

Other than GAC, several low-cost and environmentally friendly sorbent materials are being explored for commercial and large-scale implementation [23]. For example, organoclays, which are clay minerals modified with organic cations, have proven to be effective in removing various types of contaminants [24,25]. Organoclays are hybrid materials formed by combining phyllosilicate clays, such as montmorillonite and kaolinite, with organic surfactants [26]. The presence of organic surfactants enhances the efficiency of clays in adsorbing organic contaminants like pesticides, herbicides, and pharmaceuticals [26]. Long-chain alkyl ammonium cations are among the typical organic surfactants used to modify the clay surface to produce organoclays. These cationic surfactants lead to the formation of a hydrophobic partition medium in the clay interlayer. The nature of the interlayer charge changes from negative to positive upon surface modification. Apart from these, the intercalation of the organic surfactants also increases the basal spacing of the clay interlayers [27]. These modifications in the clay structure make it suitable for binding PFAS compounds on its surface as they are both anionic and amphiphilic. Recent studies have highlighted the effectiveness of materials with amine functional groups in removing anionic PFAS through counterionic interactions [28]. Although numerous studies have explored PFAS removal by amine-functionalized sorbents, only a handful have been tested at environmentally relevant PFAS concentrations, where amine-functionalized sorbents such as poly(ethylenimine)-functionalized cellulose and amine-functionalized covalent organic frameworks were studied for their PFAS removal efficiency in batch experiments [28,29]. The infrared (IR) analysis highlighted the role of electrostatic interaction between the amine functional group and the PFAS sorbate in the sorption of PFAS on the sorbent surface. For example, an IR analysis of an amine-functionalized chitosan sorbent before and after PFOS adsorption showed a shift in the N-H stretch peak from 3429 cm^−1^ to 3470 cm^−1^ and a shift in the N-H bending band from 1649 cm^−1^ to 1635 cm^−1^. Organoclays, sharing many of these properties, hold significant potential for PFAS sorption, warranting further investigation. Also, there is limited research on the PFAS removal efficiency of organoclays under environmentally relevant neutral pH conditions using adsorption isotherm experiments.

In this study, we investigated the performance of commercially available GAC (Filtrasorb-400), organoclay (OC-200), and modified organoclays (Fluoro-sorb-100 and Fluoro-sorb-200) in the removal of three long-chain PFAS compounds, polyfluorooctanoic acid (PFOA), polyfluorooctane sulfonic acid (PFOS), and perfluorononanoic acid (PFNA). These PFAS compounds were selected based on the screening of PFAS contamination in groundwater from a local Superfund site. The results on sorption kinetics, sorption isotherms, and desorption behavior of PFAS compounds on commercially available reactive media will offer critical insights into the effectiveness and potential limitations of these sorbents for PFAS remediation and provide guidance for the selection of appropriate media in future real-world field applications, e.g., being used as reactive media in PRBs for contaminated groundwater treatment.

## 2. Materials and Methods

### 2.1. Materials and Characterization

The PFAS compounds, PFOA, PFOS, and PFNA, used in this study were procured from Sigma Aldrich (St. Louis, MI, USA) and were of analytical grade. The four commercially available sorbent materials, OC-200, Fluoro-sorb-100, Fluoro-sorb-200, and Filtrasorb-400, were provided by Tetra Tech Inc. (King of Prussia, PA, USA).

X-ray diffraction (XRD) analysis was conducted to identify the crystalline phases and composition of the sorbent materials using Rigaku Ultima IV (Wilmington, MA, USA). To assess the surface morphology and elemental composition of each sorbent, scanning electron microscopy (SEM) coupled with energy-dispersive spectroscopy (EDS) was performed using a Zeiss FIB SEM, equipped with an EDS detector from Oxford Instruments (Concord, MA, USA). The surface area and pore size of the sorbents were determined via nitrogen adsorption–desorption isotherms using an ASAP2020 surface area analyzer from Micromeritics Instrument Corporation (Norcross, GA, USA). Fourier transform infrared spectra (FTIR) were obtained using an ATR-FTIR spectrometer (Bruker, Billerica, MA, USA) to surface-characterize the sorbent materials for chemical functional groups in the mid-IR wavenumber region (400–4000 cm^−1^).

### 2.2. Sorption and Desorption Experiments

Laboratory-scale adsorption isotherm and kinetic experiments were conducted to evaluate the sorption behavior of PFOA, PFOS, and PFNA on the four commercially available sorbents, OC-200, Fluoro-sorb-100, Fluoro-sorb-200, and Filtrasorb-400. Adsorption isotherm experiments were performed at a neutral pH of 6.8 ± 0.2 and a dosage of 0.8 g sorbents in 40 mL of 10 mM NaNO_3_ solution following Zhang et al. [8]. Initial PFAS concentrations were set at 100, 300, 500, 700, 1000, 2000, 3000, and 5000 µg/L. Control experiments without the addition of sorbents were carried out under the same conditions. An adsorption kinetic study was performed for PFOA, PFOS, and PFNA at an initial concentration of 700 µg/L. Samples were collected at specific time points at 0.0, 0.2, 0.5, 1.0, 1.5, 2.0, 4.0, 6.0, and 9.0 h.

Following the adsorption experiment, complete phase separation between the sorbent and the aqueous phase was achieved by centrifugation at 3500× *g* for 20 min using Eppendorf 5804 centrifuge (Hamburg, Germany). The aqueous phase was decanted and 40 mL of 0.1 mM NaNO_3_ was added to the PFAS-laden sorbent. The initial pH of the suspension was set to 6.8 ± 0.2. The reactors were agitated for 24 h at 150 rpm and 20 °C on the end-over-end shaker. After 24 h, the supernatant was collected for analysis to quantify the amount of PFAS released from the PFAS-laden sorbents. All the experiments were conducted at 20 °C in duplicate.

### 2.3. PFAS Quantification

Quantitative analysis of PFOA, PFNA, and PFOS was conducted using a Waters Micromass Quattro Ultima quadrupole mass spectrometer (Milford, MA, USA) coupled with an Agilent 1100 HPLC system. An Xterra MS C18 analytical column (150 mm × 4.6 mm; 5.0 μm particle size, from Waters (Milford, MA, USA)) and a Phenomenex delay column (50 mm × 4.6 mm; 3.0 μm particle size, from Phenomenex (Torrance, CA, USA)) were used. The instrument was operated in ESI negative mode, and the data were acquired in multiple reaction monitoring mode.

### 2.4. Data Analysis

The adsorption capacities of different sorbents across various PFAS concentration levels were assessed by analyzing the experimental data using Langmuir and Freundlich isotherm models. According to the Langmuir isotherm model, sorbate molecules do not interact with each other, and the sorption process occurs in a localized monolayer [30]. In contrast, the Freundlich isotherm model can describe multilayer adsorption where interaction between sorbate molecules is considered. Kinetics of PFOS, PFOA, and PFNA sorption on all four sorbents was modeled by employing pseudo-first-order and pseudo-second-order kinetics [31]. More information about the Langmuir and Freundlich isotherm models and the adsorption kinetics models can be found in the Supplementary Information (SI).

## 3. Results and Discussion

### 3.1. Sorbent Characterization

**Figure 1:**
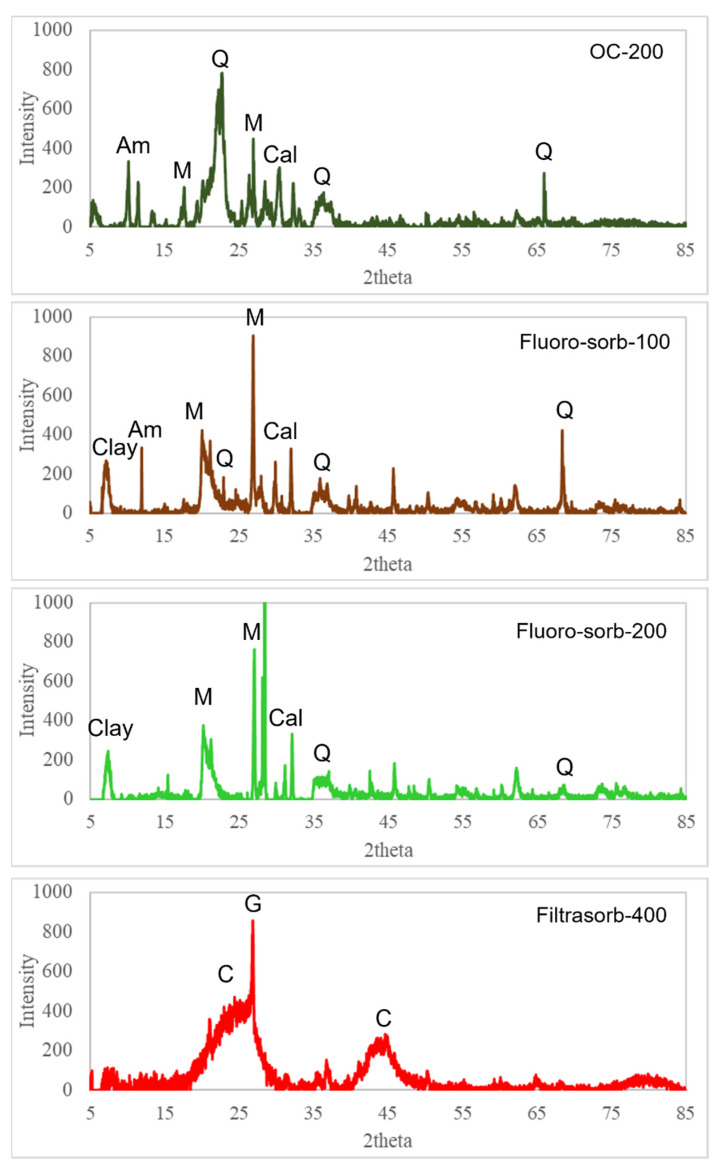
XRD spectra of sorbents used in the study. Note: M = montmorillonite, Q = quartz, Cal = calcite, Am = ammonium salt, C = amorphous carbon, G = graphite, Clay = clay layer basal spacing.

XRD spectra of the sorbents used in the study are demonstrated in Figure 1. OC-200 exhibited characteristic peaks of montmorillonite at 2θ values of 19.5° and 26.4°, along with strong quartz peaks at 20.2°, 36.2°, and 66.06° and a distinct calcite peak at 29.4° [32]. Additionally, the peak at 10.1° suggests the presence of quaternary ammonium salt [33]. Quaternary ammonium salts are used in the synthesis of organoclays. Hence, their XRD signatures are expected in OC-200. Fluoro-sorb-100 and -200 exhibited similar XRD patterns, with a prominent peak at 7.19°, corresponding to the basal spacing of the clay layers [34]. Additionally, the presence of characteristic quartz peaks was noted, which could be because of impurity. Filtrasorb-400 exhibited two broad peaks at 24° and 43.5°, characteristic of the amorphous structure of activated carbon, and the presence of a small but sharp peak at 26.55°, suggesting traces of graphite crystals [35,36].

**Figure 2:**
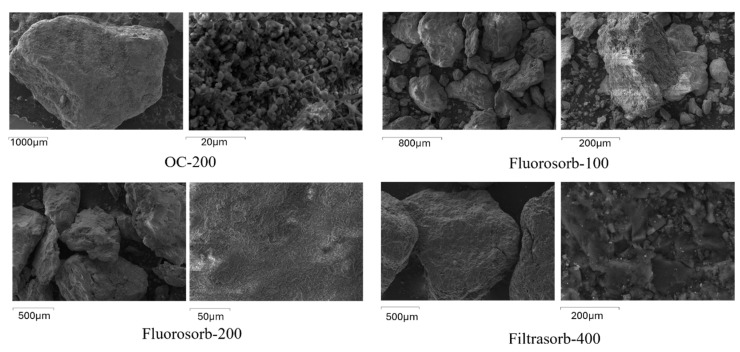
SEM images of sorbents OC-200, Fluoro-sorb-100, Fluoro-sorb-200, and Filtrasorb-400 indicated a range of different surface morphologies and particle sizes.

The surface morphology of Filtrasorb-400 exhibited larger particles with numerous ridges and pores (Figure 2), which is consistent with observations reported in other studies [36]. Due to this porous structure, Filtrasorb-400 has a large specific surface area as compared to other sorbents, and, hence, a large number of surface sites are available for non-specific sorption. Fluoro-sorb-100 and Fluoro-sorb-200 exhibited similar rough morphologies with fewer visible pores compared to Filtrasorb-400, with Fluoro-sorb-100 having a smaller average particle size (Figure 2). It should be noted that Fluoro-sorb-100 and Fluoro-sorb-200 differ in their average particle size (Table S1). They have similar bond structures and, hence, comparable sorption characteristics. In contrast, OC-200 showed a significantly larger particle size distribution, with most particles in the millimeter range. The surface characteristics of OC-200 were similar to those of Fluoro-sorb-100 and Fluoro-sorb-200.

The EDS elemental composition data indicated that OC-200, Fluoro-sorb-100, and Fluoro-sorb-200 are primarily composed of silicon, carbon, and oxygen (Table S2), further confirming the dominance of montmorillonite and quartz in these sorbents, as supported by the XRD analysis. As anticipated, Filtrasorb-400, being an activated carbon sorbent, was predominantly composed of carbon. Although all these sorbents effectively removed various PFAS species, the fluorine peak was only detected in Filtrasorb-400, OC-200, and Fluoro-sorb-100, rather than Fluoro-sorb-200 after the adsorption experiments (Table S2). This is likely due to the low surface concentration of PFAS and the varying adsorption mechanisms of each sorbent. For example, clay-based sorbents may sequester PFAS within the interlayers of the clay sheets rather than on the material’s surface [34]. The example EDS mapping of the PFAS-bearing sorbents is shown in Figure S1.

The specific surface area of the sorbents was measured by employing the BET technique (Table 1). All three organoclays showed similar specific surface areas, while the granular activated carbon, Filtrasorb-400, exhibited a significantly higher specific surface area, possibly due to its highly porous nature. Notably, this increased surface area did not correspond to higher PFAS removal rates, suggesting that other factors, such as the presence of functional groups, may influence the adsorption extent. Additionally, the pore size of Filtrasorb-400 may be insufficient for the internal diffusion of PFAS species, leading to primary adsorption on the surface and leaving the internal surface area inaccessible for PFAS molecules.

### 3.2. Sorption Kinetics

**Figure 3:**
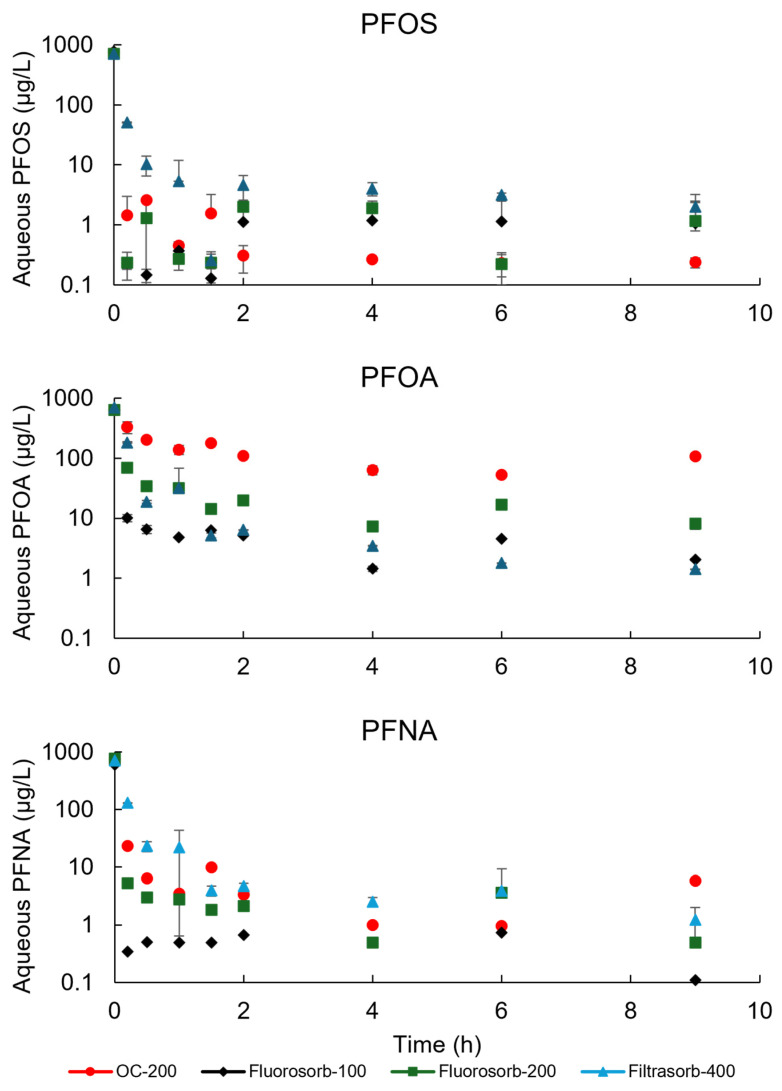
Kinetics of PFNA, PFOS, and PFOA adsorption on sorbents. Y-axis is on logarithmic scale.

The sorption kinetics of four sorbents for the PFOA, PFOS, and PFNA were assessed for a period of 9 h. Nearly all of the initially introduced PFAS were rapidly adsorbed by the sorbents within minutes (Figure 3), independent of their estimated particle size distributions [37]. The observed rapid adsorption kinetics for PFAS are indicative of a high affinity and abundance of available adsorption sites, which promote efficient PFAS removal through hydrophobic and electrostatic mechanisms [7,38]. PFNA and PFOS exhibit rapid initial adsorption, reaching plateau rates within 0.2 h, regardless of types of sorbents; however, PFOA exhibits a more gradual approach to equilibrium, with a continuing increase in adsorption capacity over time for organoclay (OC-200) (Figure 3). In previous studies, PFOS sorption on an organically modified montmorillonite clay and an oleylamine-modified palygorskite clay (MatCARE^TM^) showed comparatively slower kinetics where the sorption equilibriums were achieved after 32 h and 1 h, respectively [39,40]. The initial PFOS concentration adopted in this work was comparatively much lower at ~700 µg/L than what was adopted in these previous works. Moreover, a direct comparison of the PFOS adsorption trends among all works will not be feasible as other factors (e.g., pH and ionic strength) may also affect the sorption process, which may explain the faster sorption equilibrium in this study.

Sorption kinetics was illustrated by fitting the experimental data to the pseudo-first- and pseudo-second-order models (Figures S2–S5). The key parameters of both models are presented in Table 2. All sorbents exhibited a superior fit to the pseudo-second-order kinetic model (R^2^ > 0.98) compared to the pseudo-first-order model, suggesting that the pseudo-second-order model more accurately describes the adsorption process. This implies chemical interaction may have been involved in the PFAS sorption process. These pseudo-second-order kinetics of PFAS sorption are in agreement with the findings in similar studies where PFAS sorption on clay minerals was studied [39,41]. The value of the pseudo-second-order rate constant k_2_ for PFOS sorption on all the four commercial sorbents studied in this work is 4 orders of magnitude higher than those observed for PFOS sorption on montmorillonite clays, demonstrating the faster kinetics of PFOS sorption on the commercial sorbents. Moreover, it also demonstrates that these sorbents have a strong capacity for PFAS removal attributed to their high density of sorption sites [42].

Out of the three PFAS compounds studied in this work, PFOS showed the highest sorption kinetics with comparatively higher k_2_ values followed by those for PFNA and PFOA. This denotes that PFOS complexation on the sorbent surface is faster than PFNA, followed by PFOA. The experimental q_e_ values (described in the Supplementary Information) were in agreement with those generated by the pseudo-second-order kinetic models for all sorbate–sorbent pairs, indicating the model’s goodness of fit.

Rapid equilibrium times for PFNA and PFOS suggest that shorter contact times may be sufficient in treatment processes, while the gradual increase in sorption for PFOA on OC-200 indicates that longer contact times might be necessary for better removal. Despite variations in the rate at which different PFAS species reached equilibrium, competitive effects likely did not influence the total amount absorbed, given that complete absorption was achieved for all PFAS chain lengths [37]. However, a study on the competitive effects would be of interest in future work.

### 3.3. Sorption Isotherms

Removal efficiency for each PFAS–sorbent pair was estimated by employing Equation (1).

(1) $$RE\left(\%\right)=\frac{\left(C_{0}-C_{t}\right)}{C_{0}}\times 100\%$$

where C_0_ and C_t_ are concentrations of the PFAS at time 0 and time t, respectively.

**Figure 4:**
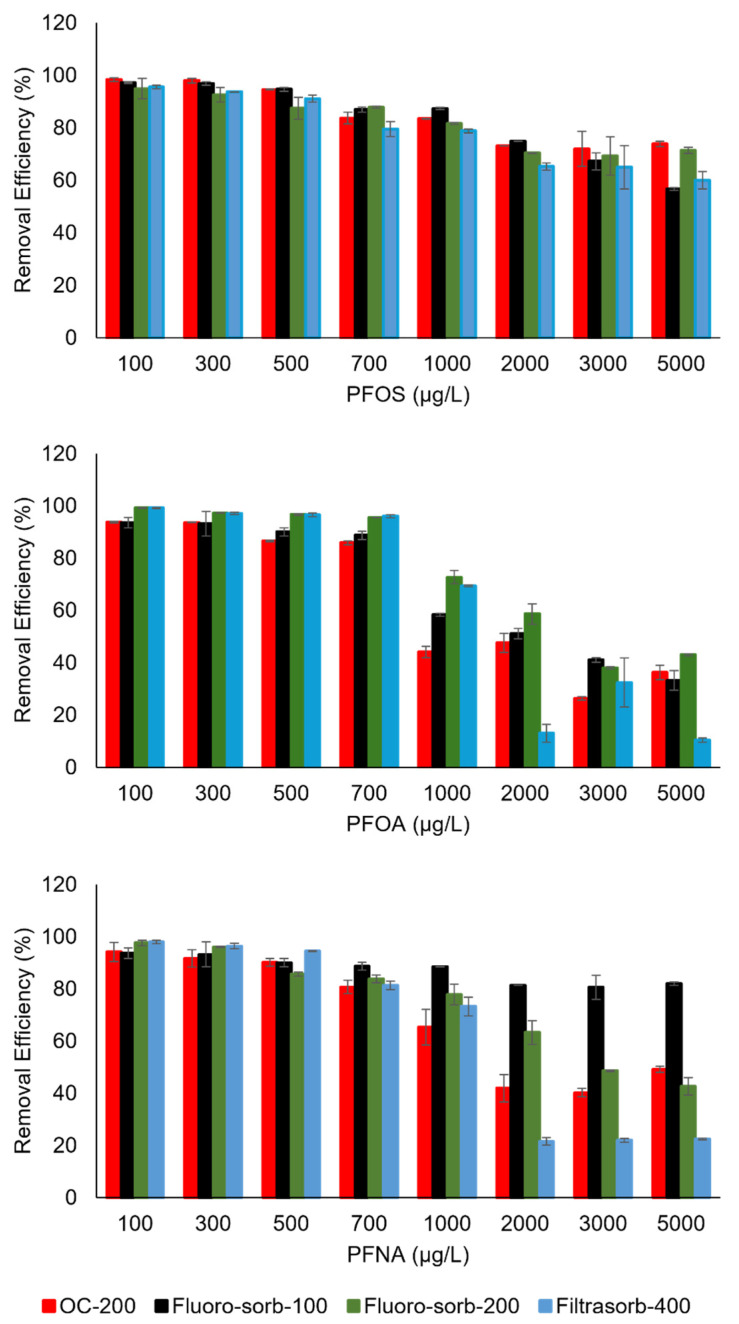
Removal efficiency of PFOS, PFOA, and PFNA by the four sorbents for different initial PFAS concentrations at pH 6.8 ± 0.2 and 20 °C.

All sorbents, GAC (Filtrasorb-400), organoclay (OC-200), and modified organoclays (Fluoro-sorb-100 and Fluoro-sorb-200) showed excellent removal efficiencies (>80%) for all three PFAS compounds at initial PFAS concentration below 700 µg/L, while the removal efficiencies dropped when initial PFAS concentrations were further increased (Figure 4). Briefly, for PFOS, all four sorbents demonstrated >60% removal at a high initial sorbate concentration of 5000 µg/L. In contrast, PFOA removal efficiencies for all sorbents were <40% at a high initial sorbate concentration of 5000 µg/L. The removal efficiencies of PFNA were between those of PFOS and PFOA, which is in agreement with their relative sorption affinity.

A study by Johnson et al. [43] on the global distribution of PFAS contamination notes that the arithmetic means of the concentration of PFOA and PFOS in all the primary source groundwater contamination sites reported globally were 64 and 93 µg/L, respectively [43]. The mean PFOA and PFOS concentrations in the secondary source contamination sites and the sites without any known source were comparatively much lower. As we observed >90% removal for an initial PFAS concentration of ~100 µg/L for all three PFAS compounds, PFOS, PFNA, and PFOA, in our work, all four sorbent materials are suitable for PFAS removal at environmentally relevant levels. In comparison, at levels of PFOA and PFNA higher than 2000 µg/L, both modified organoclays (i.e., Fluoro-sorb-100 and Fluoro-sorb-200) outperformed GAC (i.e., Filtrasorb-400), which is probably due to the electrostatic interactions between anionic PFOA and PFNA and modified organoclays’ cationic functional groups [44]. This is consistent with Murray et al. [44], where modified organoclays performed better than GAC in removing the tested perfluoroalkyl acids (PFAAs) and perfluoroalkane sulfonic acids (PFSAs) from water. However, it should be noted that the presence of other anionic contaminants, ligands, and dissolved organic matter in contaminated groundwater can significantly impact the removal efficiencies of PFAS compounds on these commercial sorbents, and, hence, future studies will be important to understand those complex interactions [45,46,47]. Furthermore, the shorter-chain PFAS compounds will likely have a weaker binding affinity as compared to the long-chain compounds and, hence, lower sorption extent. In a real-world scenario, over a certain period of time, long-chain PFAS compounds might break up to form short-chain PFAS, eventually affecting the PFAS removal efficiency of the sorbents.

The Langmuir and Freundlich isotherm models were employed to quantify the maximum adsorption capacity of each sorbent–sorbate pair (Figures S6–S9). Table 3 presents the isotherm parameters calculated from the experimental data using the Langmuir and Freundlich models. While both models yielded excellent fits (high R^2^ values), the Langmuir model was found to be marginally superior in describing the adsorption behavior of PFOA, PFNA, and PFOS, suggesting a monolayer adsorption mechanism on the sorbent surface [48,49]. This was in line with the findings of similar adsorption isotherm modeling works for PFOS adsorption on surface-modified clay minerals [40,50].

The maximum observed adsorption capacity, q_m_, which is the maximum amount of adsorbate that can be adsorbed per unit mass of the adsorbent, for PFOS for the organoclays/modified organoclays ranged from ~55 to 65 µg/g. For comparison, the q_m_ of PFOS on oleylamine-modified palygorskite was ~700 times higher than what we estimated for the organoclay minerals studied in this work [40]. This comparatively high sorption capacity can be attributed to either the stronger affinity of the PFOS on the oleylamine-modified palygorskite surface or other background conditions that can control sorption capacity, like pH. Although palygorskite is a 2:1 phyllosilicate, unlike smectites, it has a needlelike structure. This makes palygorskite poorer in specific surface area as compared to smectites, and, hence, it has a smaller sorption capacity as compared to smectites. All organoclays and modified organoclays we studied in this work were composed of montmorillonite, as evidenced by the XRD analysis (Figure 1). Therefore, all of them have superior sorption capacity as compared to the oleylamine-modified palygorskite at a given background condition. Moreover, the cation exchange capacity of palygorskite is also smaller as compared to smectites (i.e., between 20 and 60 meq/100 mL), again supporting the fact that the sorption capacity of surface-modified palygorskite will be lower than the surface-modified smectites at a given background condition. However, it should be noted that the equilibrium pH adopted in their work was ~5.00, which is almost 2 pH units lower than what was employed in this work. Hence, a higher sorption capacity of PFOS in their work as compared to ours indicates the importance of pH in determining the sorption affinity; more PFOS is adsorbed at lower pH because of electrostatic interactions when the surface is positively charged. Among the sorbents evaluated, organoclays and modified organoclays showed better PFAS removal performance compared to GAC. In addition, the modified organoclays, Flouro-sorb-100, showed the highest q_m_ value for PFOS and PFNA, while the organoclay OC-200 showed the greatest capacity for PFOA removal. The q_m_ values for PFOS were higher than those for PFNA and PFOA, indicating a stronger affinity of the sorbents for PFOS.

**Figure 5:**
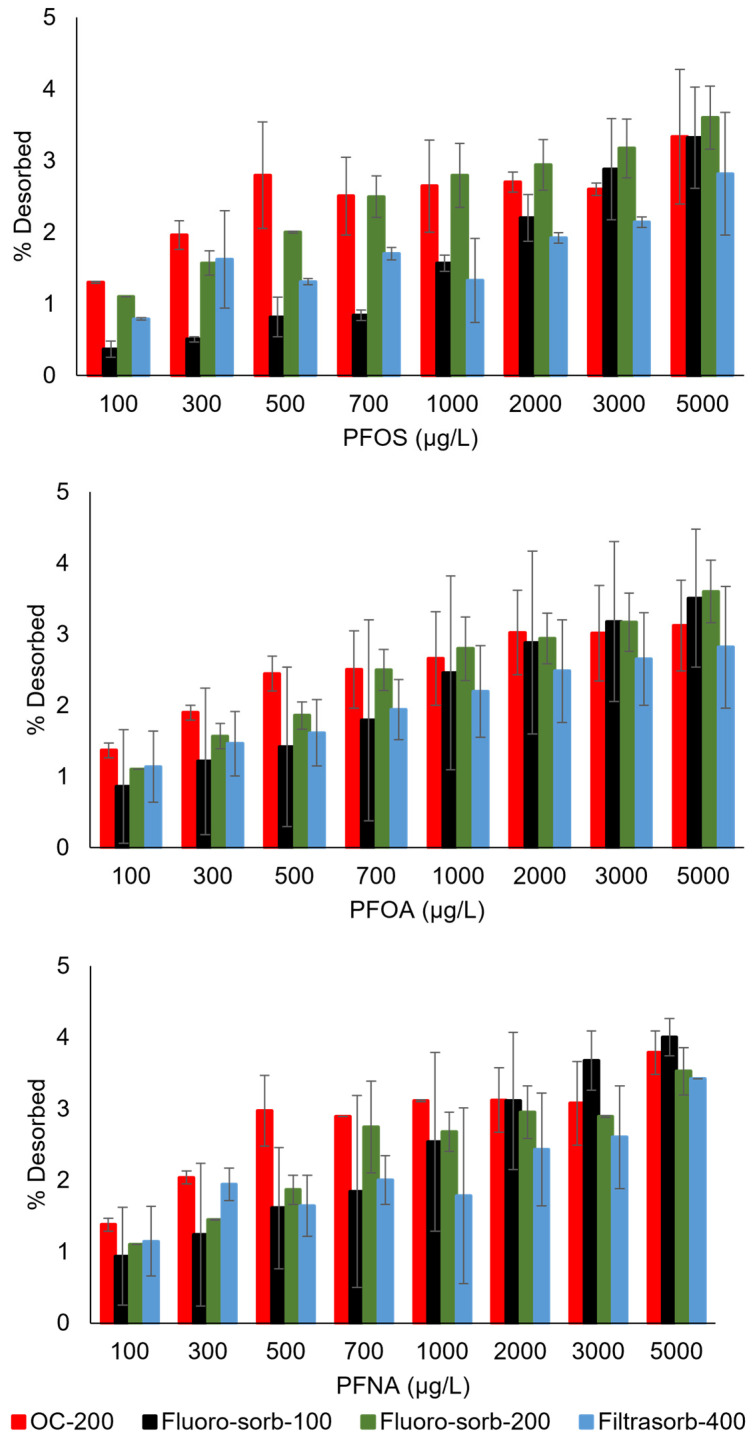
Percentage of PFAS desorbed from the sorbents at pH 6.8 ± 0.2 and 20 °C.

The desorption profiles of PFOA, PFOS, and PFNA for various sorbents were evaluated under controlled conditions (pH 6.8 ± 0.2) across the initial concentration range of 100–5000 µg/L (Figure 5). Notably, the desorbed fractions of PFOS and PFNA remained below 10% of the total adsorbed mass for most sorbent materials across all initial concentrations. PFOA desorption was generally lower, with values typically under 5% for all sorbents. Filtrasorb-400 and Flouro-sorb-200 demonstrated superior performance by consistently exhibiting the lowest desorption fractions for all three PFAS compounds across the entire PFAS concentration range. Low desorption fractions of the PFAS compounds denote that sorption is largely irreversible and, hence, possibly dominated by inner-sphere complexation [37]. Low desorption extent further highlights the suitability of all these commercial sorbents for in situ field application, such as in the PRBs for groundwater remediation.

Clay minerals have two types of sites on which sorbates can bind: (a) edge sites, which behave similarly to the sorption sites present in (oxy)hydroxide minerals, and (b) interlayer cation exchange sites, which are typically permanently negatively charged. The surface charge on the edge sites is a direct function of the pH, and, hence, any sorption on edge sites is a strong function of pH. On the other hand, the surface charge on the interlayer cation exchange sites is independent of pH, and the availability of the cation exchange sites is a strong function of the ionic strength. The surface charge on the edge sites will also be affected. In organoclays, the interlayer basal space is occupied by organic ammonium cations, leading to the neutralization of the negative surface charge that was present in the clay before its surface modification. Therefore, most of the sorption on organoclay surfaces is expected to take place on the edge sites. Unlike the sorption on the cation exchange sites, the edge site sorption is inner-sphere in nature and, hence, irreversible. This is further supported by the findings of the desorption experiments, where we observed minimal desorption of PFAS sorbates.

### 3.4. Potential PFAS Interactions with Sorbents

The sorbent materials were characterized using FTIR spectroscopy, in the mid-IR region (400–4000 cm^−1^). Several characteristic vibrational regions pertaining to functional groups that may play a crucial role in the adsorption processes of PFAS compounds could be identified using FTIR spectroscopy.

**Figure 6:**
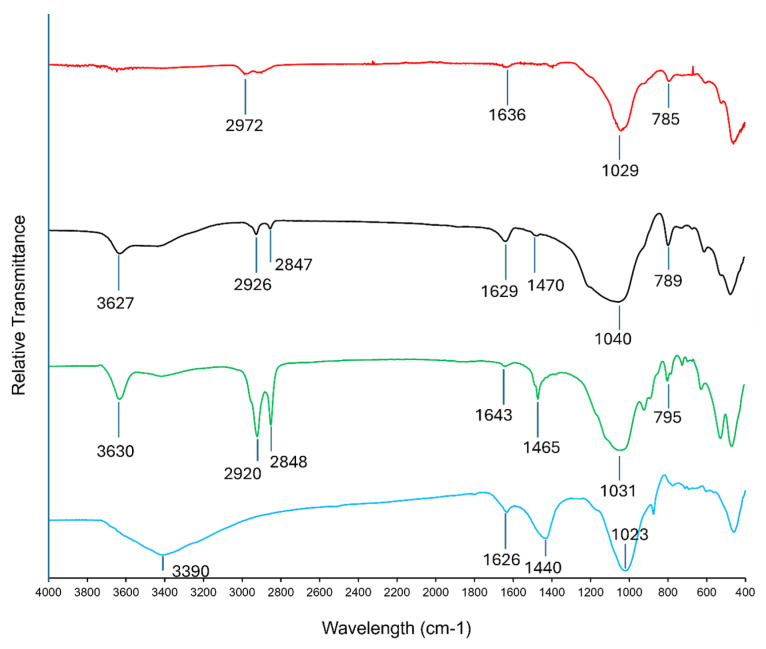
FTIR spectra overlay of OC-200 (red), Fluoro-sorb-100 (black), Fluoro-sorb-200 (green), and Filtrasorb-400 (blue).

Many prominent IR spectral features can be observed in the FTIR spectra of the tested sorbent materials (Figure 6). A broad peak typically appearing around 3200-3600 cm^−1^ can be associated with the stretching vibrations of hydroxyl groups (–OH) [51], which was prominently identified in Fluoro-sorb-100 and Fluoro-sorb-200. This indicated the presence of surface hydroxyl groups or adsorbed water molecules on Fluoro-sorb-100 and Fluoro-sorb-200, which was expected in organoclay-based sorbents. PFAS anions can bind onto those surface hydroxyl sites due to electrostatic interactions [52,53].

Peaks around 2800–3000 cm^−1^ corresponded to the stretching vibrations of C-H bonds in aliphatic or aromatic structures [54]. These can be identified as prominent spectral features in the IR spectra of OC-200, Fluoro-sorb-100, and Fluoro-sorb-200. These peaks suggested the presence of residual hydrocarbons or surface-bound organic molecules. PFAS can bind to these aliphatic groups due to strong hydrophobic interactions [55].

A strong peak around 1650–1750 cm^−1^ is indicative of carbonyl functional groups [51]. They are prominent in the activated carbon-based sorbent material, Filtrasorb-400. These groups could be part of carboxylic acids, esters, or ketones formed during the activation process of the GAC-based sorbent materials such as Filtrasorb-400. Additional peaks in the range of 1600–1650 cm^−1^ and 1300–1400 cm^−1^ can be attributed to the stretching vibrations of carboxylate and lactone groups, respectively [56]. These surface oxides contribute to the acidic nature of the activated carbon surface. Peaks occurring around 1500–1600 cm^−1^ in FTIR spectra are typically associated with the stretching vibrations of aromatic C=C bonds [51]. This indicates that the carbon structure of Filtrasorb-400 contains aromatic rings, characteristic of the graphitic nature of activated carbon. Peaks in the region of 700–900 cm^−1^ correspond to the out-of-plane bending vibrations of C-H bonds in aromatic rings, further confirming the presence of aromatic structures [57]. Filtrasorb-400 typically showed peaks at about 3400, 2400, 1630, 1380, 1180, and 460 cm^−1^, which could be mostly associated with surface functional groups containing oxygen (i.e., O–H, C=O, and C–O). PFAS compounds are expected to bind to these GAC surface sites mostly due to hydrophobic interactions [58,59,60].

## 4. Conclusions

The selection of suitable reactive media for the removal of PFAS from contaminated water is of critical importance. Two types of commercial sorbents, granular activated carbon (Filtrasorb-400) and organoclays (OC-200, and modified organoclays Fluoro-sorb-100 and Fluoro-sorb-200), were tested in this study for the sorption of PFOS, PFOA, and PFNA. All four sorbents studied showed high removal efficiencies for PFAS compounds at an initial concentration of <700 µg/L and at a near-neutral, environmentally relevant pH of 6.8 ± 0.2, with organoclays typically outperforming GAC in terms of sorption capacity. Desorption experiments revealed low PFAS release from all sorbents, suggesting an essentially irreversible adsorption process. The excellent removal efficiencies, rapid sorption kinetics, and low desorption observed for all four commercial sorbents at environmentally relevant pH demonstrated their high potential in water treatment applications where the fast and efficient removal of contaminants is paramount. Although this study incorporates many environmentally relevant background conditions like pH, ionic strength, initial PFAS concentration, and sorbent concentration, the presence of other competing anions, both organic and inorganic, in water was not accounted for. Similarly, the presence of hydrophobic organics in the water can also impact the sorption of the PFAS compounds, which was also not tested. Future research should focus on evaluating the sorption performance of these sorbents under conditions encountered in real wastewater and groundwater environments.

## Figures and Tables

**Table 1:** BET surface area and average pore size of the sorbents.

Sorbent	Specific Surface Area (m^2^/g)	Micropore Volume (cm^3^/g)	Total Pore Volume (cm^3^/g)	Average Pore Size (nm)
OC-200	106	<LOD	0.20	6.69
Fluoro-sorb-100	92	<LOD	0.08	4.54
Fluoro-sorb-200	113	<LOD	0.10	4.45
Filtrasorb-400	827	0.23	0.56	4.53

Note: LOD = Limit of Detection.

**Table 2:** Kinetic parameters for PFOA, PFOS, and PFNA adsorption on sorbents.

Sorbent	Sorbate	Pseudo-First Order		Pseudo-Second Order		
		R^2^	k_1_ (hr^−1^)	R^2^	q_e_ (µg/g)	k_2_ (g/µg-hr)
OC-200	PFOA	0.46	0.17	0.99	27.7	0.43
	PFOS	0.31	0.46	1.00	36.5	25.0
	PFNA	0.24	0.32	1.00	38.0	34.5
Fluoro-sorb-100	PFOA	0.29	0.31	1.00	31.6	9.99
	PFOS	0.02	0.11	1.00	38.5	67.6
	PFNA	0.20	0.38	1.00	30.4	54.1
Fluoro-sorb-200	PFOA	0.42	0.28	0.99	31.1	1.47
	PFOS	0.06	0.19	1.00	35.1	40.6
	PFNA	0.29	0.38	1.00	38.2	13.7
Filtrasorb-400	PFOA	0.60	0.53	1.00	34.8	1.03
	PFOS	0.21	0.33	1.00	35.2	4.03
	PFNA	0.55	0.50	1.00	35.7	1.31

**Table 3:** Langmuir and Freundlich isotherm parameters for PFOA, PFOS, and PFNA on sorbents.

Sorbent	Sorbate	Langmuir Constants			Freundlich Constants		
		R^2^	$q_{m}(µg/g)$	$K_{L}$	R^2^	n	$K_{F}$
OC-200	PFOS	0.979	55.56	0.056	0.952	2.116	4.399
	PFOA	0.973	47.17	0.087	0.818	2.846	3.872
	PFNA	0.990	47.62	0.018	0.912	2.344	3.020
Fluoro-sorb-100	PFOS	0.990	65.79	0.027	0.974	2.110	3.983
	PFOA	0.990	22.12	0.16	0.924	2.561	3.557
	PFNA	0.995	99.01	0.0080	0.991	1.399	1.386
Fluoro-sorb-200	PFOS	0.984	58.82	0.017	0.987	1.679	1.927
	PFOA	0.961	10.81	0.52	0.913	3.202	7.533
	PFNA	0.973	43.29	0.052	0.983	2.448	4.093
Filtrasorb-400	PFOS	0.985	54.95	0.021	0.979	1.916	2.515
	PFOA	0.906	25.45	0.29	0.369	6.998	10.62
	PFNA	0.982	32.15	0.089	0.703	4.149	7.272

Note: MSE data for both isotherm models can be found in Table S3.

## Data Availability

The original contributions presented in this study are included in the article/supplementary material. Further inquiries can be directed to the corresponding author.

## Supplementary Materials

The following supporting information can be downloaded at https://www.mdpi.com/article/10.3390/ma18061299/s1.

## Abbreviations

The following abbreviations are used in this manuscript:

EDS	Energy-dispersive spectroscopy
FTIR	Fourier transform infrared spectra
GAC	Granular activated carbon
PFAAs	Perfluoroalkyl acids
PFAS	Per- and polyfluoroalkyl substance
PFNA	Perfluorononanoic acid
PFOA	Polyfluorooctanoic acid
PFOS	Polyfluorooctane sulfonic acid
PFSAs	Perfluoroalkane sulfonic acids
SEM	Scanning electron microscopy
XRD	X-ray diffraction
