# Supplementary material for: Removal of Per- and Polyfluoroalkyl Substances Using Commercially Available Sorbents
Source: Materials (Basel). 2025 Mar 15;18(6):1299. doi: 10.3390/ma18061299 (PMC11943809; doi:10.3390/ma18061299)
Supplement: Supplementary file 1 [file materials-18-01299-s001.zip › materials-3499418-supplementary.pdf]

Supplementary Information

Removal of per- and polyfluoroalkyl substances by commercially available sorbents

Zhiming Zhang[1,$], Sevda Joudiazar[2,$], Anshuman Satpathy[2], Eustace Fernando[2], Roxana Rahmati[2], Junchul Kim[3], Giacomo de Falco[4], Rupali Datta[5], and Dibyendu Sarkar [2,*]

[1]*Department of Civil and Environmental Engineering, Rowan University, Glassboro, NJ 08028, USA*

[2]*Department of Civil, Environmental and Ocean Engineering, Stevens Institute of Technology, Hoboken, NJ 07030, USA*

[3]*Tetra Tech, Inc., King of Prussia, PA 19406, USA*

[4]*New York City Department of Environmental Protection, New York City, NY 11368, USA*

[5]*Department of Biological Sciences, Michigan Technological University, Houghton, MI 49931, USA*

[*] *Corresponding authors: dsarkar@stevens.edu*

The adsorption capacities of different sorbents across various PFAS concentration levels were assessed by analyzing the experimental data using Langmuir and Freundlich isotherm models.

The Langmuir isotherm is described in Equation S1.

$$\frac{1}{q_e} = \left(\frac{1}{q_{max}K_L}\right)\frac{1}{C_e} + \frac{1}{q_{max}} \tag{S1}$$

Where, q_e is the adsorption capacity at chemical equilibrium (µg/g), K_L (L/ µg) is Langmuir's isotherm constant; q_{max} is the maximum adsorption capacity (µg/g).

The Freundlich model is described in Equation S2.

$$lnq_e = \frac{1}{n}C_e + lnK_F \tag{S2}$$

Where K_f is Freundlich's constant; and 1/n is the adsorption intensity.

Kinetics of PFOS, PFOA, and PFNA sorption on all four sorbents was modeled by employing pseudo first order and pseudo-second-order kinetics. The equations describing those kinetics models are shown in Equations (S3) and (S4), respectively.

$$\ln\left(\frac{C_t}{C_0}\right) = k_1 t \tag{S3}$$

Where C_0 is the initial aqueous concentration of the analyte, C_t is the aqueous concentration of the analytes after time t and k_1 is the pseudo first-order rate constant.

$$\frac{t}{q_t} = \frac{1}{q_e} \cdot t + \frac{1}{k_2} \cdot \frac{1}{q_e^2} \tag{S4}$$

Where q_e is the sorption capacity at equilibrium, q_t is the sorption capacity at time t and k_2 is the pseudo second-order rate constant.

Table S1. Particle sizes of the four commercially available sorbent materials

Materials	Particle Size Description
OC-200	600-1850 micron
Filtrasorb-400	0.55 to 0.75 mm
Fluoro-sorb-100	The material has a broad particle size distribution, with most particles around 0.45 mm, 92% passing through a 0.85 mm mesh, and 8% smaller than 0.075 mm.
Fluoro-sorb-200	The material has a relatively uniform particle size distribution, with most particles around 0.64 mm, 93% passing through a 0.85 mm mesh, and 95% of the material larger than 0.36 mm.

Table S2. EDS elemental composition of (a) OC-200, (b) Filtrasorb-400, (c) Fluoro-sorb-100, (d) Fluoro-sorb-200 before PFAS adsorption, and (e) OC-200, (f) Filtrasorb-400, (g) Fluoro-sorb-100, (h) Fluoro-sorb-200 after PFAS adsorption.

Element	Weight(%)
C	22.63±0.18
O	41.16±0.14
Na	0.65±0.02
Mg	0.39±0.02
Al	4.84±0.03
Si	25.13±0.09
P	0.14±0.02
S	0.11±0.02
Cl	1.43±0.03
K	0.95±0.03
Ca	1.12±0.04
Fe	1.44±0.14

(a)

Element	Weight(%)
C	20.27±0.15
O	44.79±0.13
F	0.46±0.05
Na	0.48±0.02
Mg	0.46±0.02
Al	5.15±0.03
Si	25.21±0.08
P	0.1±0.02
S	0.04±0.02
K	0.72±0.03
Ca	0.43±0.03
Fe	1.88±0.15

(e)

Element	Weight(%)
C	92.96±0.07
O	4.69±0.06
Al	0.69±0.01
Si	0.93±0.02
S	0.72±0.02

(b)

Element	Weight(%)
C	89.79±0.08
O	5.47±0.07
F	1.85±0.05
Na	0.09±0.01
Al	0.86±0.02
Si	1.1±0.02
S	0.84±0.02

(f)

Element	Weight(%)
C	32.33±0.24
N	1.07±0.18
O	39.56±0.27
Na	0.3±0.05
Mg	0.88±0.06
Al	5.77±0.1
Si	15.02±0.17
Fe	5.07±0.37

(c)

Element	Weight(%)
C	29.58±0.23
N	1.24±0.17
O	38.48±0.26
F	0.08±0.13
Na	0.13±0.04
Mg	0.77±0.05
Al	6.39±0.1
Si	17.15±0.18
Fe	6.18±0.37

(g)

Element	Weight(%)
C	25.78±0.3
N	0.83±0.22
O	36.99±0.34

Element	Weight(%)
C	28.32±0.2
N	1.06±0.15
O	39.84±0.22

Na	1.12±0.08
Mg	1.01±0.08
Al	7.67±0.16
Si	20.86±0.27
Fe	5.73±0.48

(d)

Na	0.13±0.04
Mg	0.83±0.05
Al	6.7±0.09
Si	16.85±0.15
Fe	6.26±0.31

(h)

Table S3. MSE information for both Langmuir and Freundlich isotherm models

Adsorbent	PFAS	Langmuir MSE	Freundlich MSE
OC-200	PFOA	24.73	9040
	PFOS	2574	414.7
	PFNA	769.9	254.4
Fluoro-sorb-100	PFOA	7.320	6379
	PFOS	980.4	22.50
	PFNA	1966	120.4
Fluoro-sorb-200	PFOA	7.000	284.6
	PFOS	2203	174.1
	PFNA	703.7	13.76
Filtrasorb-400	PFOA	18.01	281.7
	PFOS	1464	62.17
	PFNA	92.55	87.56

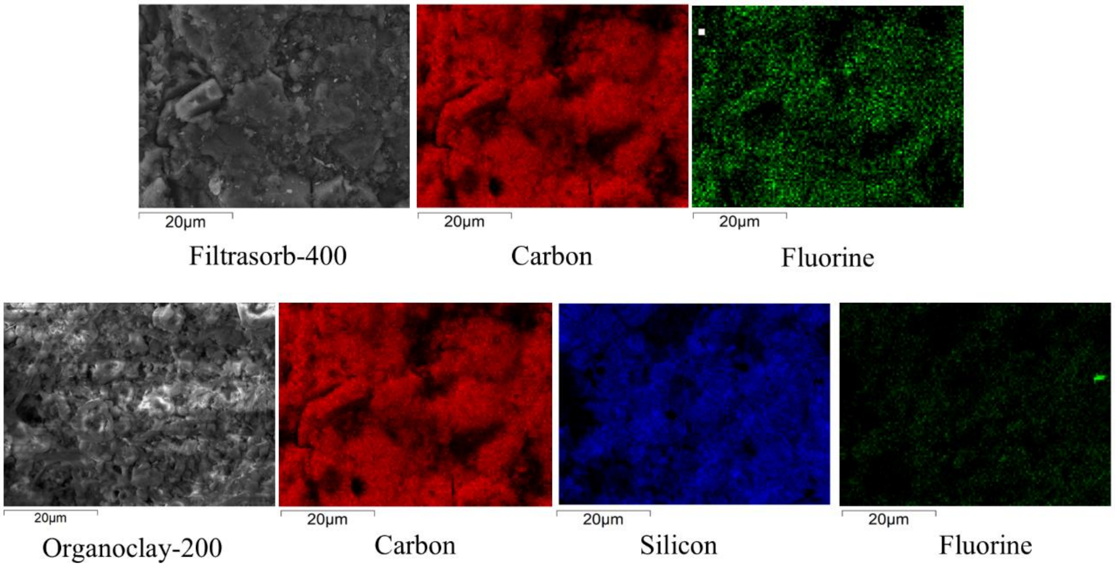

Figure S1. EDS spectra of Filtrasorb-400 and OC-200 after adsorption of PFOS.

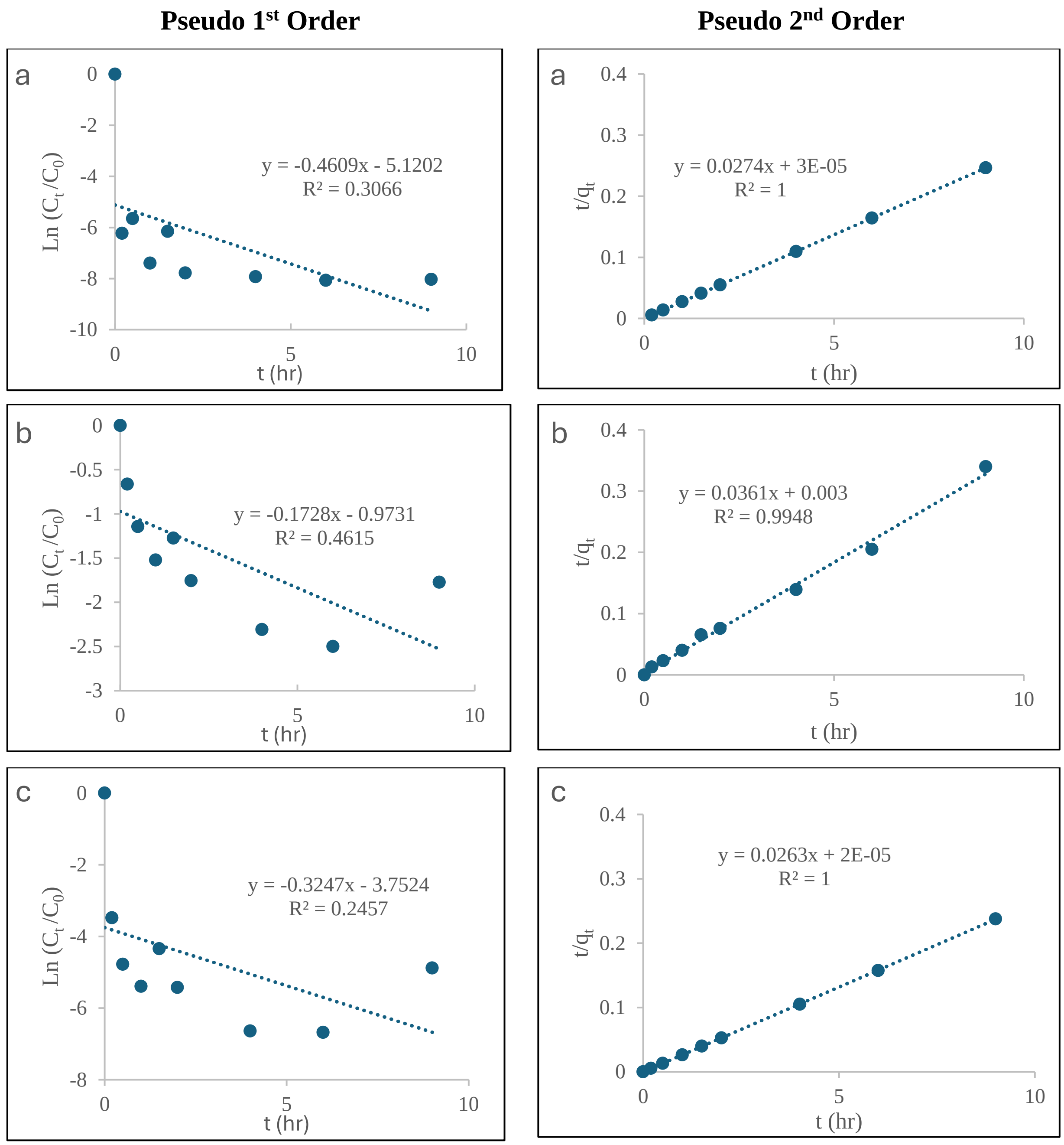

Figure S2. Pseudo 1st and 2nd order kinetic plots for the adsorption of a) PFOS, b) PFOA, and c) PFNA on OC-200

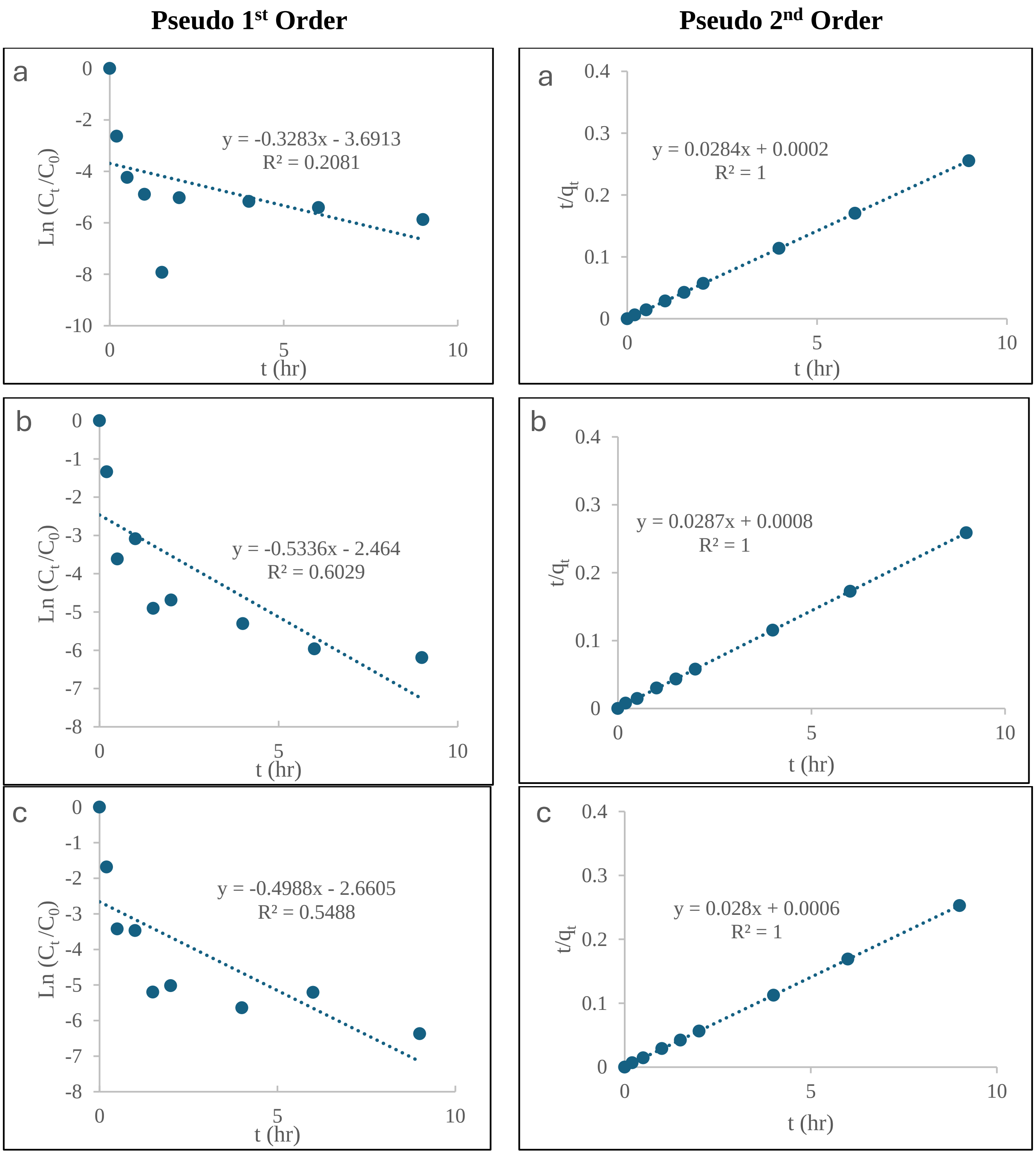

Figure S3. Pseudo 1st and 2nd order kinetic plots for the adsorption of a) PFOS, b) PFOA, and c) PFNA on Filtrasorb-400

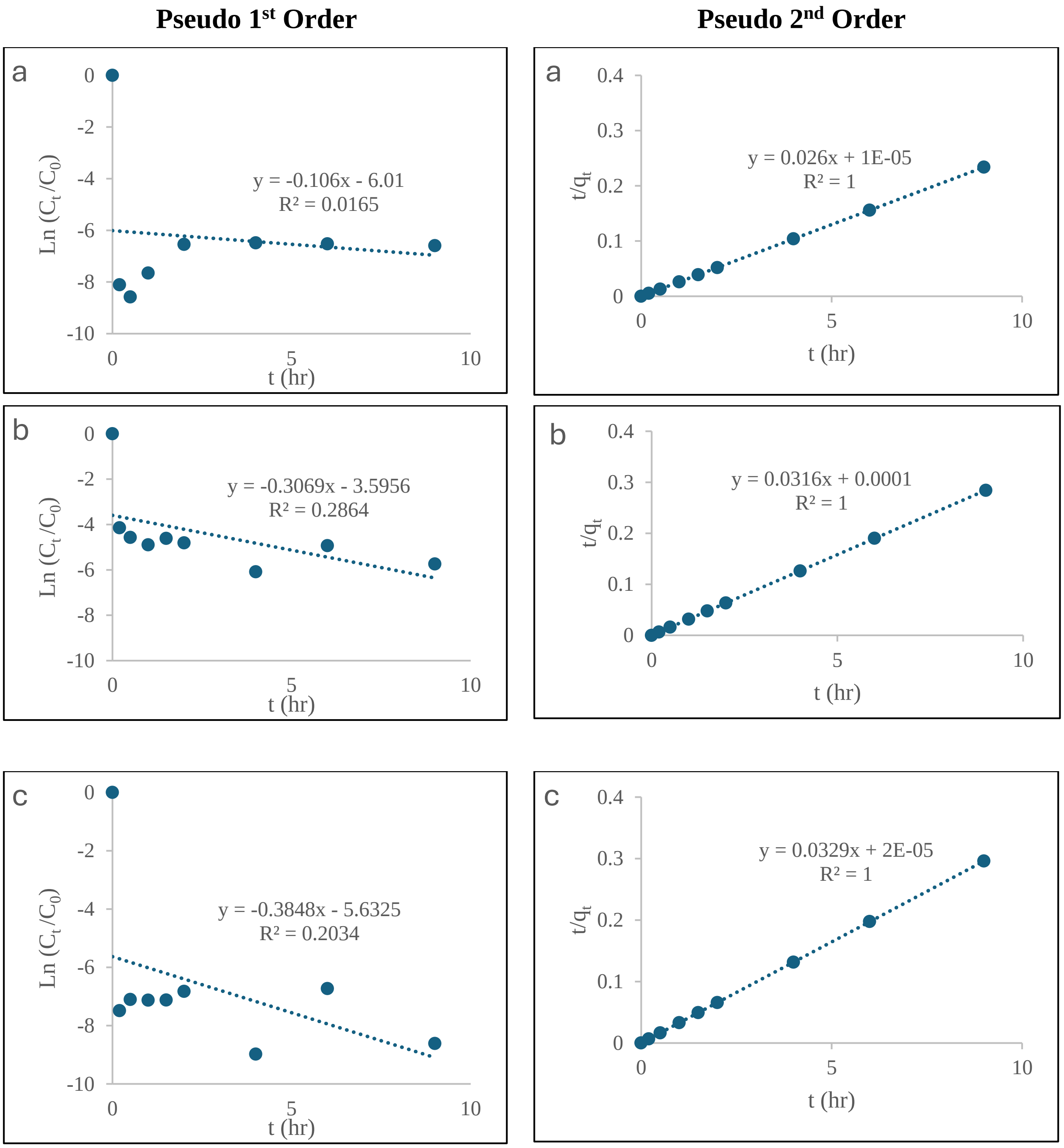

Figure S4. Pseudo 1st and 2nd order kinetic plots for the adsorption of a) PFOS, b) PFOA, and c) PFNA on Fluoro-sorb-100

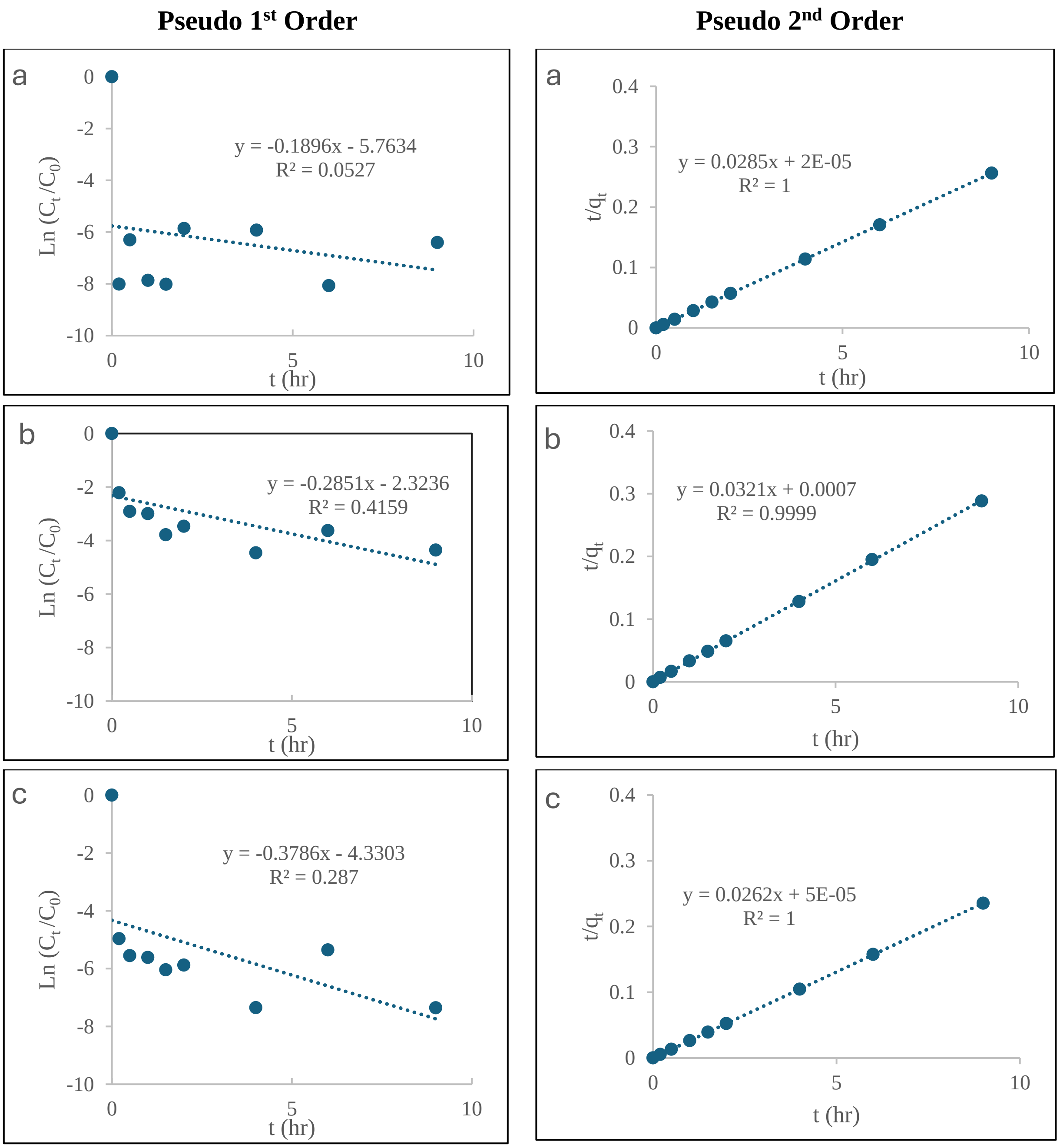

Figure S5. Pseudo 1st and 2nd order kinetic plots for the adsorption of a) PFOS, b) PFOA, and c) PFNA on Fluoro-sorb-200

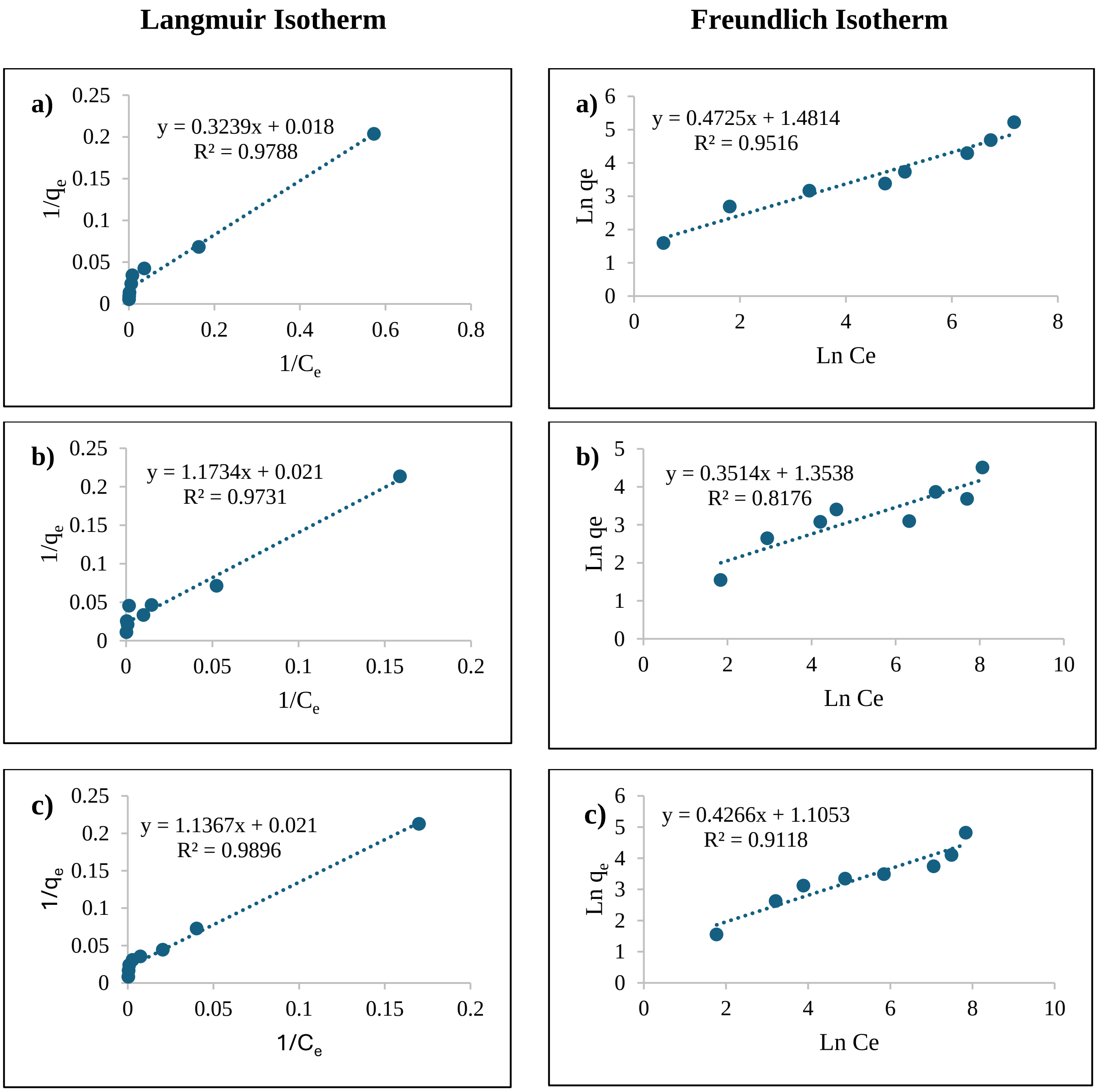

Figure S6. Langmuir and Freundlich isotherm models for the adsorption of a) PFOS, b) PFOA, and c) PFNA on OC-200

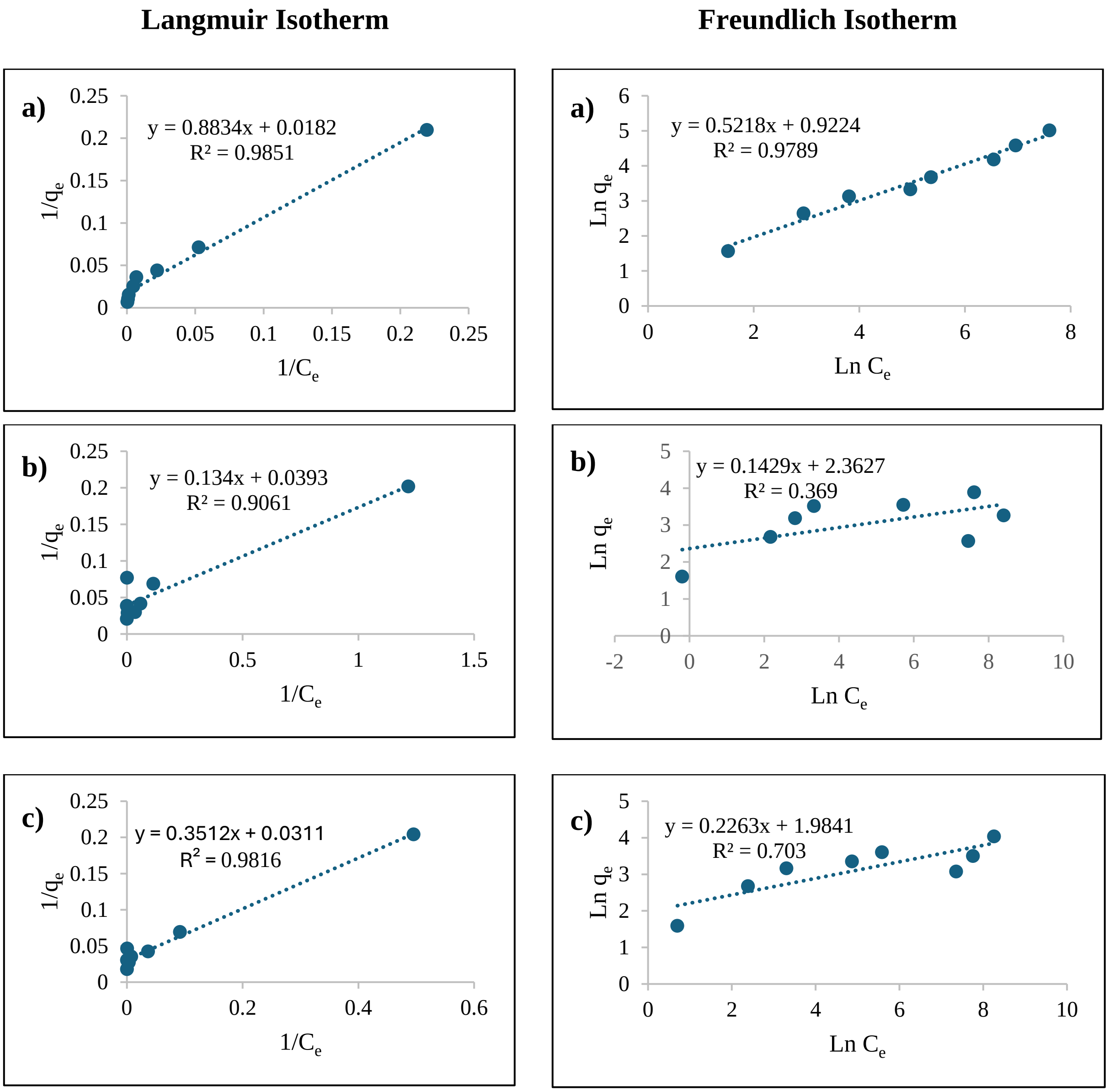

Figure S7. Langmuir and Freundlich isotherm models for the adsorption of a) PFOS, b) PFOA, and c) PFNA on Filtrasorb-400

Langmuir Isotherm **Freundlich Isotherm**

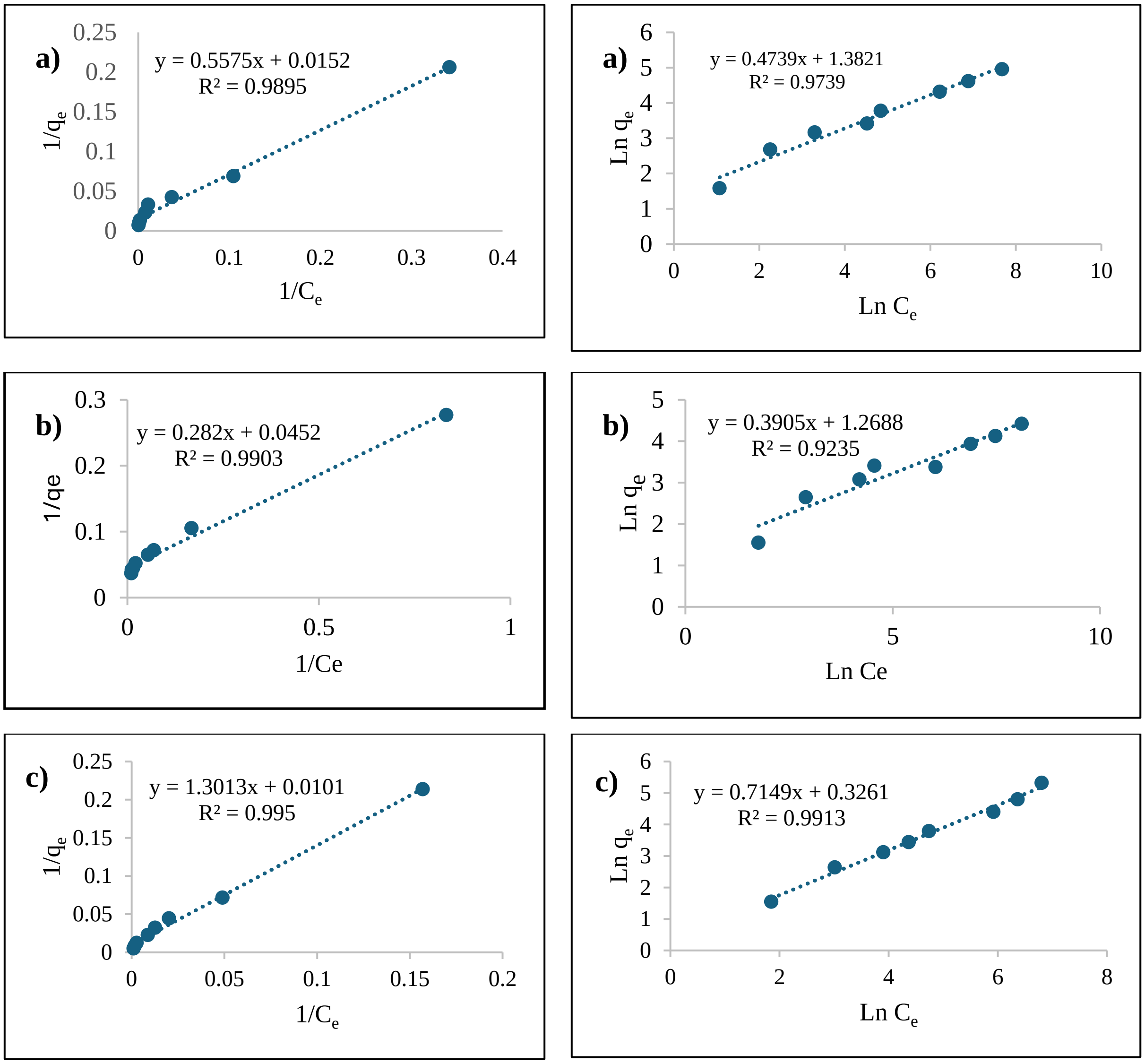

Figure S8. Langmuir and Freundlich isotherm models for the adsorption of a) PFOS, b) PFOA, and c) PFNA on Fluoro-sorb-100

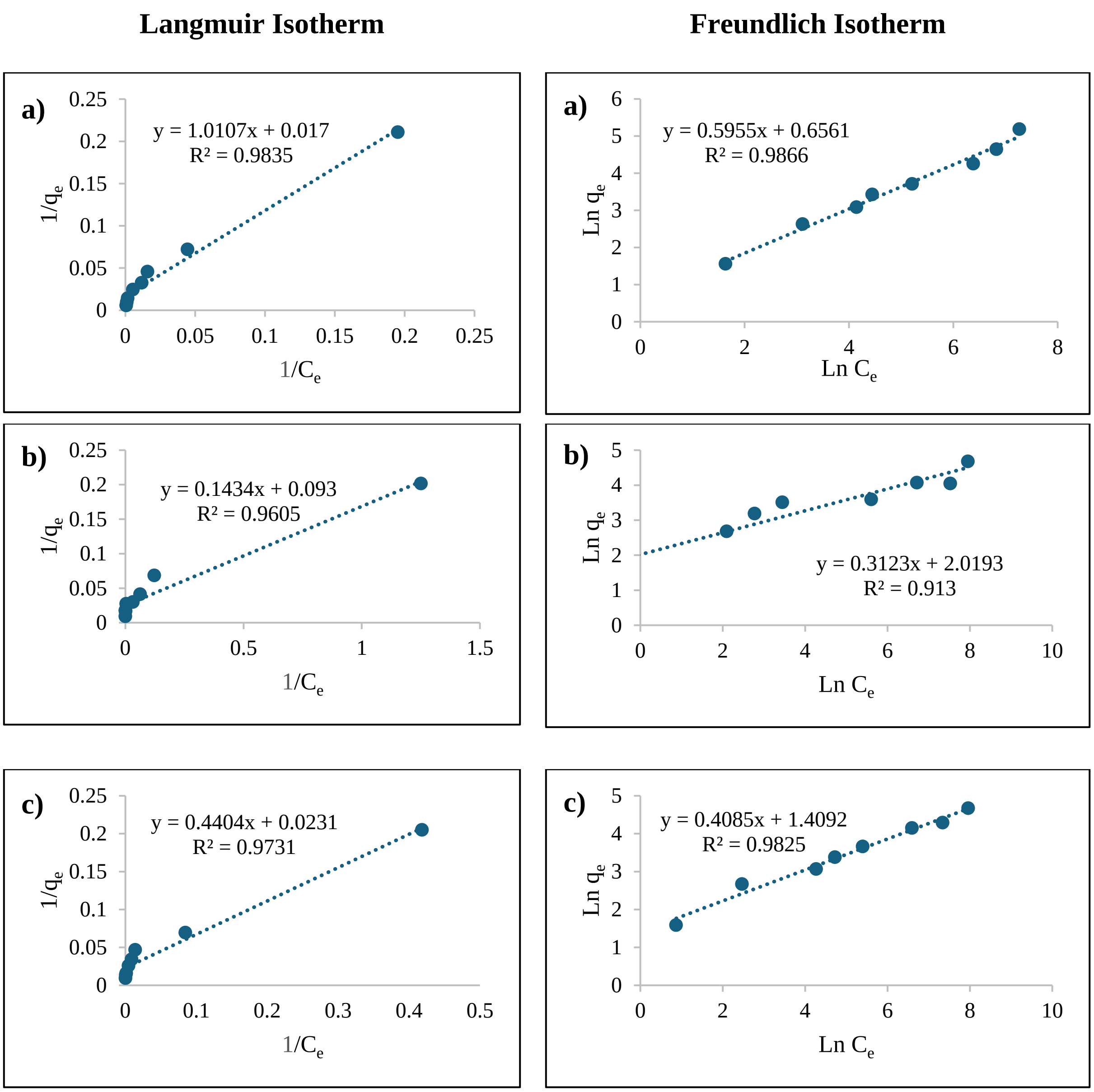

Figure S9. Langmuir and Freundlich isotherm models for the adsorption of a) PFOS, b) PFOA, and c) PFNA on Fluoro-sorb-200
